# Anatomical effects of ablation of the anterior retina in small animal eyes

**DOI:** 10.1186/s12886-026-04818-5

**Published:** 2026-04-15

**Authors:** Jiayun Wang, Tibor Lohmann, Yuli Wu, Kim Schaffrath, Henning Konermann, Kaan Keven, Frederic Balcewicz, Sandra Johnen, Johannes Stegmaier, Peter Walter, Sabine Baumgarten

**Affiliations:** 1 Department of Ophthalmology, Uniklinik RWTH Aachen, Pauwelsstrasse 30, 52074 Aachen, Germany; 2 Institute of Imaging and Computer Vision, RWTH Aachen University, Kopernikusstrasse 16, 52074 Aachen, Germany; 3 Present Address: Faculty of Mathematics and Natural Sciences, Heinrich Heine University Düsseldorf, Universitätsstrasse 1, 40225 Düsseldorf, Germany

**Keywords:** Retinal coagulation, Ophthalmologic surgery, Choroid-retinal adhesion, Animal model

## Abstract

**Background:**

To provide anatomical data after cryo and laser ablation of the anterior retina in mice, rats, and rabbits as a prerequisite to make surgical procedures in these species safer.

**Methods:**

12 eyes per species of C57BL/6J wild-type (*wt*) mice, Brown Norway rats, and Chinchilla Bastard rabbits, respectively, were treated with laser or cryo coagulation in the anterior retina. Diode laser and cryo coagulation were applied transsclerally. The coagulation effect was evaluated histologically after three weeks and a three-dimensional (3D) reconstruction was performed from serial microscopic sections to improve visualization of the effects.

**Results:**

Lesion diameter differed significantly between cryo and laser coagulation across all species (911.61 ± 52.59 μm vs. 775.89 ± 56.11 μm, *p* < 0.05 for mice, 686.44 ± 42.98 μm vs. 519.19 ± 30.59 μm, *p* < 0.0001 for rats, 415.32 ± 28.43 μm vs. 313.65 ± 29.35 μm, *p* < 0.05 for rabbits). The ratio of retinal thickness before and after cryo and laser coagulation was 0.41 ± 0.01 and 0.50 ± 0.03 for mice, 0.46 ± 0.03 and 0.51 ± 0.01 for rats, and 0.45 ± 0.01 and 0.51 ± 0.01 for rabbits, respectively. No significant side effects were observed. Using segmentation and registration algorithms, the microscopic images of each stack were aligned and 3D visualized.

**Conclusions:**

Cryo pretreatment of the anterior retina provided a broader-based scarring compared to laser pretreatment and can possibly help to optimize experimental vitreoretinal procedures to test the biocompatibility and function of prototype devices for retinal stimulation.

## Background

New vitreoretinal surgical techniques are often tested in rabbits or even in rats or mice. Small animal species are easier to keep and care for in an animal facility when compared e.g. to pigs, dogs, or cats. However, due to the lack of a pars plana the placement of sclerotomies, e.g. for the insertion of instruments or implants, can be a major risk factor for intra- or postoperative retinal detachments. In our experience, the rate of retinal breaks or detachments in such experimental procedures can be up to 50% or more [1, 2]. Emerging concepts for retinal implants included an even higher degree of complexity as with the Argus II or Alpha IMS/AMS system: New implants will be larger than previous ones to provide a larger field of vision, and also multifunctional implants with integrated electronics for e.g. cell specific closed-loop stimulation paradigms are being developed [3–6]. This indicates that further research is required to enhance the safety of the implant and the surgical procedure.

Because the main risk factor for developing a retinal detachment in small animals’ eyes is the lack of a pars plana combined with a very anterior position of the ora serrata, we aimed at a better understanding of local effects of cryo or laser ablation of the anterior retina.

In this study, we performed cryo and laser pretreatment in the anterior retina in eyes of mice, rats, and rabbits, and evaluated the results histologically. We collected data on the size of the resulting scars to estimate how much treatment is necessary to provide stable scars for a certain incision to be safe.

We also reconstructed the anatomy and visualized it in 3D. Visualizing original stacks of serial microscopic sections through the whole eye is challenging due to artifacts, positional changes, background noise, and tissue damage due to histological processing. On the other hand, a correct 3D reconstruction of the treated eye may be beneficial for using such data sets in surgical simulations and in teaching.

## Methods

### Animals

Animals used within the study were 12 *wt* C57BL/6J mice (female, 9–10 weeks; Janvier, Le Genest-Saint-Isle, France), 12 Brown Norway rats (female, 13–14 weeks; Charles River, Châtillon, France) and 12 Chinchilla Bastard rabbits (female, 17–18 weeks; Charles River, Châtillon, France). Animals of each species were randomly divided into two groups: cryo (*n* = 6) and laser coagulation (*n* = 6). The animals were maintained in a 12:12 h light/dark rhythm at the Institute of Laboratory Animal Science (Faculty of Medicine, RWTH Aachen University, Aachen, Germany) with free access to water and food. Only the right eye received treatment, while the left eye served as a control. Three mice (two after cryo coagulation and one after laser) had to be excluded from the subsequent analysis due to anesthesia-related complications, and eyes of six rabbits (three after cryo and laser, respectively) had to be excluded, as serial sections of whole eyes showed significant artifacts probably due to processing-related tissue reactions, e.g., fixation and dehydration. All animal experiments were performed according to the ARVO declaration for the use of animals in research, the German Law for the Protection of Animals as well as the guidelines of FELASA after approval was obtained by the Institute of Laboratory Animal Science of the RWTH Aachen University (70017G) and the LANUK (North Rhine-Westphalia Office of Nature, Environment and Consumer Protection) (81-02.04.2021.A295).

### Anesthesia

Mice were anesthetized via inhalation with 1.75 vol% isoflurane (Forene^®^, AbbVie, Wiesbaden, Germany). Rats received 5 vol% isoflurane initially, followed by 2 vol% for 5–6 min. For surgery in Chinchilla Bastard rabbits, 4–5 mg per kg bodyweight xylazine (Xylazin 2% Bernburg^®^, Medistar, Ascheberg, Germany) and 50 to 70 mg per kg bodyweight ketamine (Ketamin 10%, Ceva Tiergesundheit GmbH, Düsseldorf, Germany) were administered initially subcutaneously and then intraveneously.

### Preliminary evaluation

Immediately prior to the treatment, a preliminary evaluation of both eyes was conducted under anesthesia. The initial assessment was performed after applying dilating eye drops containing phenylephrine hydrochloride 2.5% and tropicamide 0.5% (MS-mydriatic eye drops, Pharmacy of the RWTH Aachen University, Germany) and then proxymetacaine hydrochloride 0.5% eye drops (Proparakain-POS, Ursapharm, Saarbrücken, Germany). The ocular condition before surgery was assessed by the use of fundoscopy and digital photography. During these consecutive exams, the animals were positioned in a prone position on the examination table. In the context of fundoscopy and digital photography with a head ophthalmoscope (OMEGA 600 Indirect Ophthalmoscope; HEINE Optotechnik GmbH & Co. KG, Gilching, Germany), it was standard practice to place the 20-diopter condensing lens (Volk Optical, Inc., Mentor, USA) at approximately 1 cm anterior to the eye. This allowed for the examination and capturing of images of the posterior segment of the eye. Each assessment lasted approximately 15 min.

### Laser coagulation

Under anesthesia, laser surgery was conducted using a transscleral laser probe (DioPexyProbe, Iridex, Mountain View, USA). We used a diode laser (810 nm; OcuLight SL diode laser, IRIS Medical, Brentwood, USA), which had a tip diameter of 600 μm. We applied the same laser coagulation settings for all species: laser energy was 250 mW and exposure time was 500 ms, creating subtle white lesions, which is located approximately 1 mm away from the limbus. With a 250 mW, 500 ms transscleral Iridex laser treatment, the estimated laser energy per unit area at the retina is approximately 0.5–1.3 J/cm² in the mouse, 0.3–0.6 J/cm² in the rat, and 0.03–0.13 J/cm² in the rabbit eye. The animals were positioned in a prone orientation on the inspection table. During laser coagulation, the eye was moistened with hydroxypropyl methylcellulose 20 mg/ml eye gel (Methocel^®^ 2%, OmniVision GmbH, Puchheim, Germany). The probe was positioned parallel to the limbus on the conjunctiva, with gentle contact and light manual pressure to ensure stable positioning and consistent energy delivery, followed by confluent limbusparallel application of 10 foci for mouse and rat eyes and 30 foci for rabbit eyes. The laser spots were then checked by indirect ophthalmoscopy using the 20-diopter condensing lens.

### Cryo coagulation

The cryo coagulation procedure was conducted using a small transscleral cryoprobe (ERBEKRYO^®^, Erbe, Marietta, USA) with a tip diameter of 900 μm under indirect ophthalmoscopy. Freezing time for each eye was 1–3 s until a clear white lesion was achieved. A temperature probe set at -80 °C was positioned approximately 1 mm away from the limbus, with gentle contact and light manual pressure to ensure stable probe positioning and consistent energy delivery. Two confluent limbus-parallel treatment spots were used for mice, three for rats, and six for rabbits. The treatment foci were sequentially positioned in a linear arrangement parallel to the limbus and merged together until the retina achieved a whitish appearance. After cryo treatment, fundoscopy was performed by employing indirect ophthalmoscopy with a 20-diopter magnification to check for any signs of retinal damage.

### Follow-up examinations

After completion of the treatment, the animals underwent daily clinical examinations to ascertain their general well-being and the absence of notable signs of inflammation. The overall state of the treated eye, body weight, general condition, and natural behavior of the animals were consistently recorded and documented. Similar to the pre-operative assessment, both eyes were re-examined under anesthetic conditions three weeks after surgery to evaluate the impact of cryo and laser coagulation. At the end of the 3-week control interval, the animals were euthanized. Mice and rats were deeply anesthetized and sacrificed with an overdose of isoflurane (Forene^®^, AbbVie, Wiesbaden, Germany). Rabbits were sacrificed with an overdose of 2mL/kg bodyweight pentobarbital-sodium (Narcoren, 160 mg/mL; Boehringer Ingelheim, Ingelheim am Rhein, Germany). Finally, eyes were enucleated for further histological study.

### Histology

Eyes were fixed in Methacarn at 4 °C for 24 h. Following dehydration, the eyes were paraffin-embedded and 3 μm thick sections were serially cut using a microtome (Slide 4003E, ptm medical, Cologne, Germany) for mouse and rat eyes, while 6 μm thick sections were made for rabbit eyes. After deparaffinization, the sections were stained with hematoxylin and eosin (HE). Serial sections of mouse and rat eyes were examined and photographed using a microscope (Leica DM6000 B with 3CCD digital camera KY-F75U; Leica Mikrosysteme Vertrieb GmbH, Wetzlar, Germany). Sections of rabbit eyes were scanned with ViewPoint Software (Trimble International, Bonn, Germany).

### Registration and 3D reconstruction

A deep learning-based segmentation model called Segment Anything Model (SAM) [7] was used to omit the background of the histological slices by performing a binary segmentation task. SAM is a state-of-the-art pre-trained foundation model for general segmentation tasks. Due to its promptable design, e.g., with points or bounding boxes, SAM can predict segmentation masks given an object of interest in a zero-shot manner.

In our experiments, we provided points at the image corners as negative point prompts and one point in the image center as a positive prompt. If the segmentation results were not satisfactory upon visual assessment, we manually provided additional points as prompts. Next, we computed rigid 2D transformations using an open-source Python library SimpleITK [8] (https://simpleitk.org/) to spatially align the binary masks produced by SAM in adjacent slices. The transformation matrices were applied to the original histological slices (RGB images). We avoided using elastic transformations to maintain the histological structures of the slices. Consequently, we could stack the aligned slices after segmentation and registration to reconstruct 3D models using the software platform Blender (version 4.2.0, https://www.blender.org) with the registered image.

### Biometry from HE stained histological sections

**Fig. 1:**
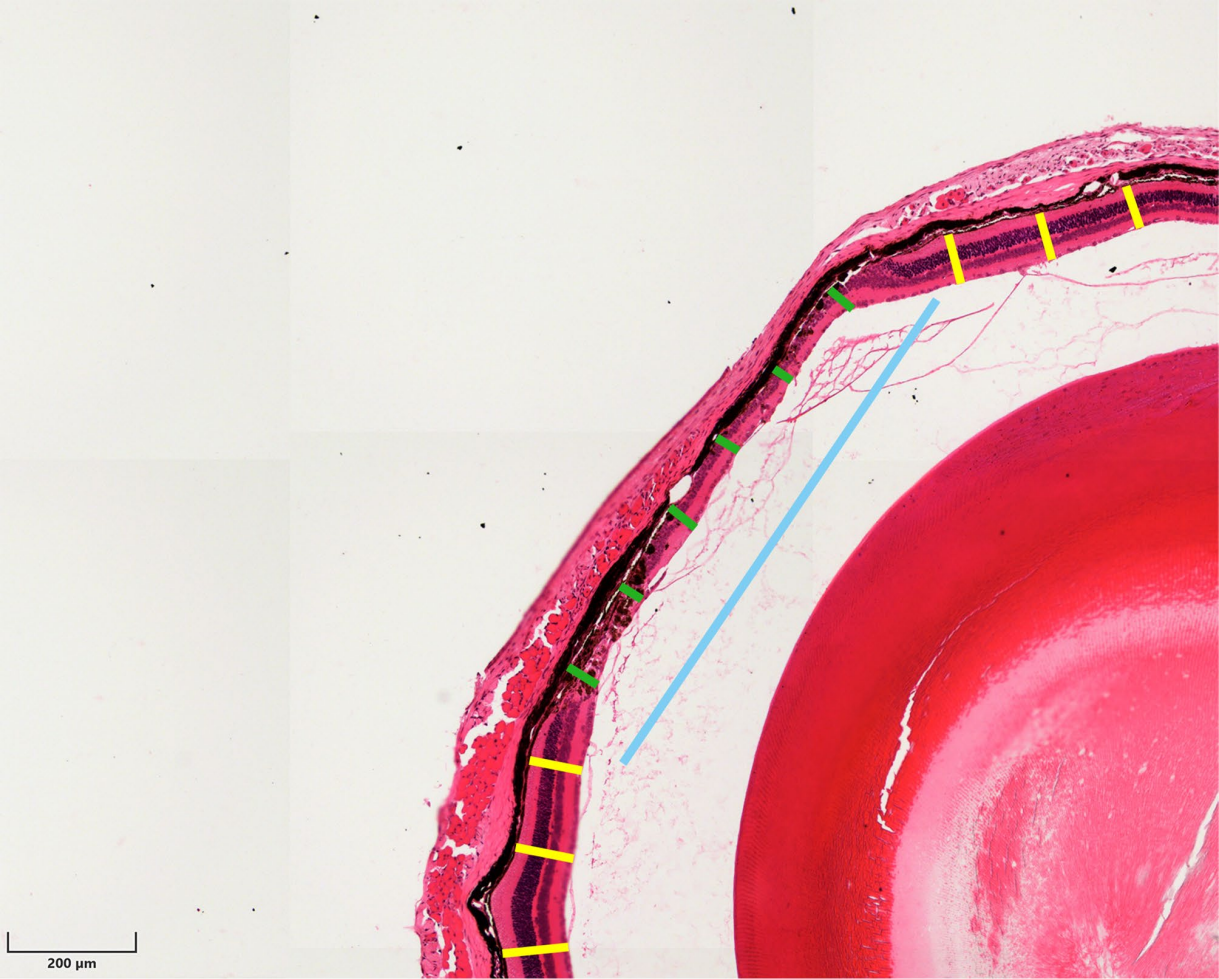
C57BL/6J mouse eye histology after laser coagulation (HE staining). Representative sagittal section illustrates the biometric parameters. Blue line: diameter of the lesion resulted by laser coagulation. Green lines: Retinal thickness evaluation at the coagulated lesion site by six evenly spaced cuts through the retina. Yellow lines: retinal thickness measurements from six uniformly distributed points within 600 μm of the scar border

The diameter of the coagulation lesions and the retinal thickness before and after cryo or laser coagulation were measured on scans of histological images. The coagulation lesions were defined as where the outer nuclear layer (ONL) appeared aberrant, and the overall retinal layers were thinned. The section showing the largest lesion diameter and clearly identifiable anatomical structures was selected for analysis. To measure retinal thickness at the site of the retinal scar, six points evenly distributed along the scar were selected for each eye (Fig. 1, green lines). Retinal thickness in non-coagulated areas was measured at six evenly spaced positions located within 600 μm from the edge of the retinal scar on cross-sectional images (Fig. 1, yellow lines). Coagulation lesions on the globe appeared as curved surfaces with arc-like diameters. However, due to their relatively tiny size, often in the range of micrometers, we approximated the area by directly measuring the diameters using straight lines (Fig. 1, blue line).

After standard histological processing, ocular tissues showed varying degrees of shrinkage, resulting in dimensions smaller than those observed in vivo. To account for dimensional changes, all morphometric data were corrected using species-specific shrinkage factors derived from our previous quantitative study [9]. In that study, shrinkage factors were calculated as the ratio of the post-processing mean to the in-vivo mean measurement for each species (shrinkage = post-processing / in-vivo). The in-vivo values were obtained from published biometric measurements, whereas post-processing values were measured from HE-stained histological sections. Lesion diameters (lateral metrics) were corrected using the axial length shrinkage factors (mouse 0.923, rat 0.816, rabbit 0.858), whereas retinal thickness (vertical metrics) was corrected using the central retinal thickness shrinkage factors (mouse 0.925, rat 0.727, rabbit 0.826). Since the retinal thickness ratio is a relative metric, no additional shrinkage correction was applied to the ratio itself. Retinal thickness was measured at multiple locations within each histological section, corrected for shrinkage, and averaged per retina to produce the recorded value. The ratio of the retinal thickness after coagulation to the retinal thickness before coagulation was calculated.

In addition, scleral thickness in the treated area was estimated from representative histological sections overlying the laser or cryocoagulation lesion. Measurements were performed perpendicular to the scleral surface on the best evaluable sections where the sclera could be clearly delineated. Because histological artifacts and section orientation occasionally limited visualization of the sclera, particularly in rabbit eyes, measurements were obtained from the most suitable sections. The resulting values were corrected for tissue shrinkage using the species-specific factors described above.

### Statistics

Statistical analyses were performed using GraphPad Prism^®^ software version 10.6.0 (GraphPad, La Jolla, CA, USA). Exploratory comparisons between Cryo and Laser groups were initially performed using unpaired two-tailed Student’s t-tests for normally distributed data with equal variances, or Welch’s t-tests when variances were unequal. To account for multiple comparisons across species and control type I error, a two-way analysis of variance (ANOVA) was then applied with species (mouse, rat, rabbit) and treatment modality (Cryo vs. Laser) as fixed factors. Post-hoc pairwise comparisons were performed using the Holm-Šídák correction. Data are presented as mean ± standard deviation (SD), and 95% confidence intervals (CI) are provided where appropriate. Statistical significance was set at *p* < 0.05. Effect sizes were calculated using Hedge’s g to account for small sample sizes.

## Results

### Cryo and laser coagulation

For rats and rabbits, all surgeries, including anesthesia, were successfully performed, and all follow-up examinations were completed as planned. In three mice, the observation period had to be terminated before the three-week interval due to systemic complications during the general anesthesia.

The energy and laser parameters were carefully chosen as described above to induce a subtle whitening of the anterior retina, so achieving the desired therapeutic outcome. Note that the lesions were visualized with slight tissue whitening.

Following cryo and laser coagulation, the cornea of treated eyes appeared transparent. There were no signs of irritation, and the animals were in good health. Cryo-scars were observed in the anterior retina. The laser-treated eyes showed a concave region in the superior half, characterized by a single visible small hollow. There were also pale patches below this region which can be used for comparison.

**Fig. 2:**
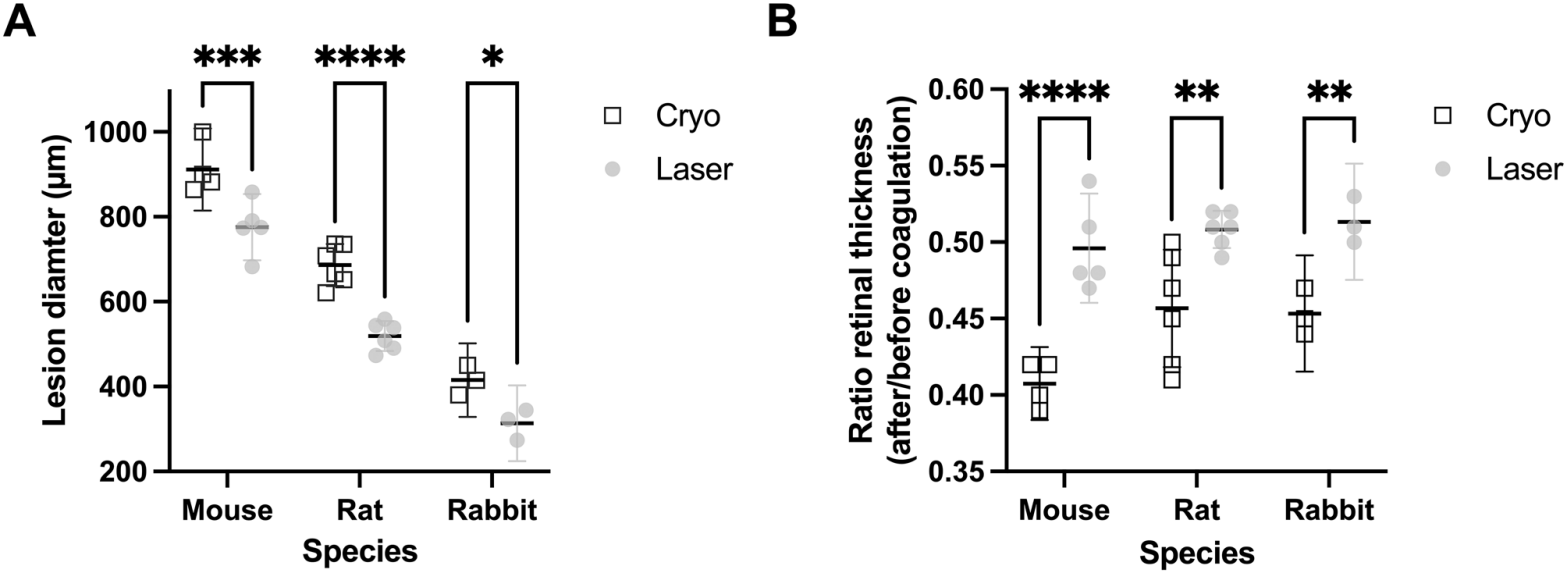
Biometric effects of cryo and laser coagulation. **A** Lesion diameter measured in C57BL/6J mice (Cryo, *n* = 4, Laser, *n* = 5), Brown Norway rats (Cryo, *n* = 6, Laser, *n* = 6), and Chinchilla Bastard rabbits (Cryo, *n* = 3, Laser, *n* = 3) three weeks after treatment. **B** Retinal thickness ratio three weeks after cryo or laser coagulation relative to pre-treatment values, plotted by species. Points represent individual animals; horizontal lines indicate group means ± 95% CI. Statistical comparisons were performed using two-way ANOVA with Holm-Šídák post hoc tests. * *p* < 0.05, ** *p* < 0.01, *** *p* < 0.001, **** *p* < 0.0001

Three weeks post-treatment, cryo coagulation caused a significantly larger lesion area and a greater retinal thickness reduction compared to laser treatment in all species, as shown in Table 1. Two-way ANOVA revealed significant main effects of species and treatment modality for both endpoints, without significant interaction. Post-hoc Holm-Šídák comparisons confirmed significant differences between cryo and laser coagulation in mice, rats, and rabbits (*p* < 0.05; Fig. 2).

Scleral thickness in the treated area was additionally estimated from representative histological sections and averaged across evaluable samples. The mean scleral thickness (± SD) was 57.46 ± 9.96 μm in mice, 109.14 ± 17.78 μm in rats, and 325.55 ± 52.60 μm in rabbits. Because these measurements were obtained from representative evaluable sections and were not systematically collected from all samples, no formal statistical comparison between treatment modalities was performed.

**Table 1:** Lesion diameters and retinal thickness ratios after Cryo versus Laser coagulation in mouse, rat, and rabbit eyes

Ocular dimensions	Mouse		Rat		Rabbit	
	Cryo (*n* = 4)	Laser (*n* = 5)	Cryo (*n* = 6)	Laser (*n* = 6)	Cryo (*n* = 3)	Laser (*n* = 3)
**Lesion diameter** (µm), mean ± SD	911.61 ± 52.59	775.89 ± 56.11	686.44 ± 42.98	519.19 ± 30.59	415.32 ± 28.43	313.65 ± 29.35
Difference between means (Laser - Cryo) ± SEM	-135.7 ± 41.51		-167.3 ± 23.59		-101.7 ± 28.90	
95% CI	-233.9 to -37.55		-219.8 to -114.7		-181.9 to -21.44	
Effect size (Hedge’s g)	-2.21		-4.14		-2.82	
*p*-value	0.0137		< 0.0001		0.0245	
**Retinal thickness ratio** (after/before), mean ± SD	0.41 ± 0.01	0.50 ± 0.03	0.46 ± 0.03	0.51 ± 0.01	0.45 ± 0.01	0.51 ± 0.01
Difference between means (Laser - Cryo) ± SEM	0.08850 ± 0.01603		0.05167 ± 0.01572		0.06000 ± 0.01247	
95% CI	0.05061 to 0.1264		0.01320 to 0.09013		0.02537 to 0.09463	
Effect size (Hedge’s g)	+ 3.39		+ 2.06		+ 4.80	
*p*-value	0.0009		0.0167		0.0086	

Differences between groups were calculated using the unpaired 2-tailed Student’s t-test or Welch’s t-test. Data are presented as mean ± standard deviation (SD). Differences between Laser and Cryo groups are shown as mean ± standard error of the mean (SEM) with 95% confidence intervals (CI), and effect sizes were calculated using Hedge’s g. P-values indicate statistical significance

### Histology

**Fig. 3:**
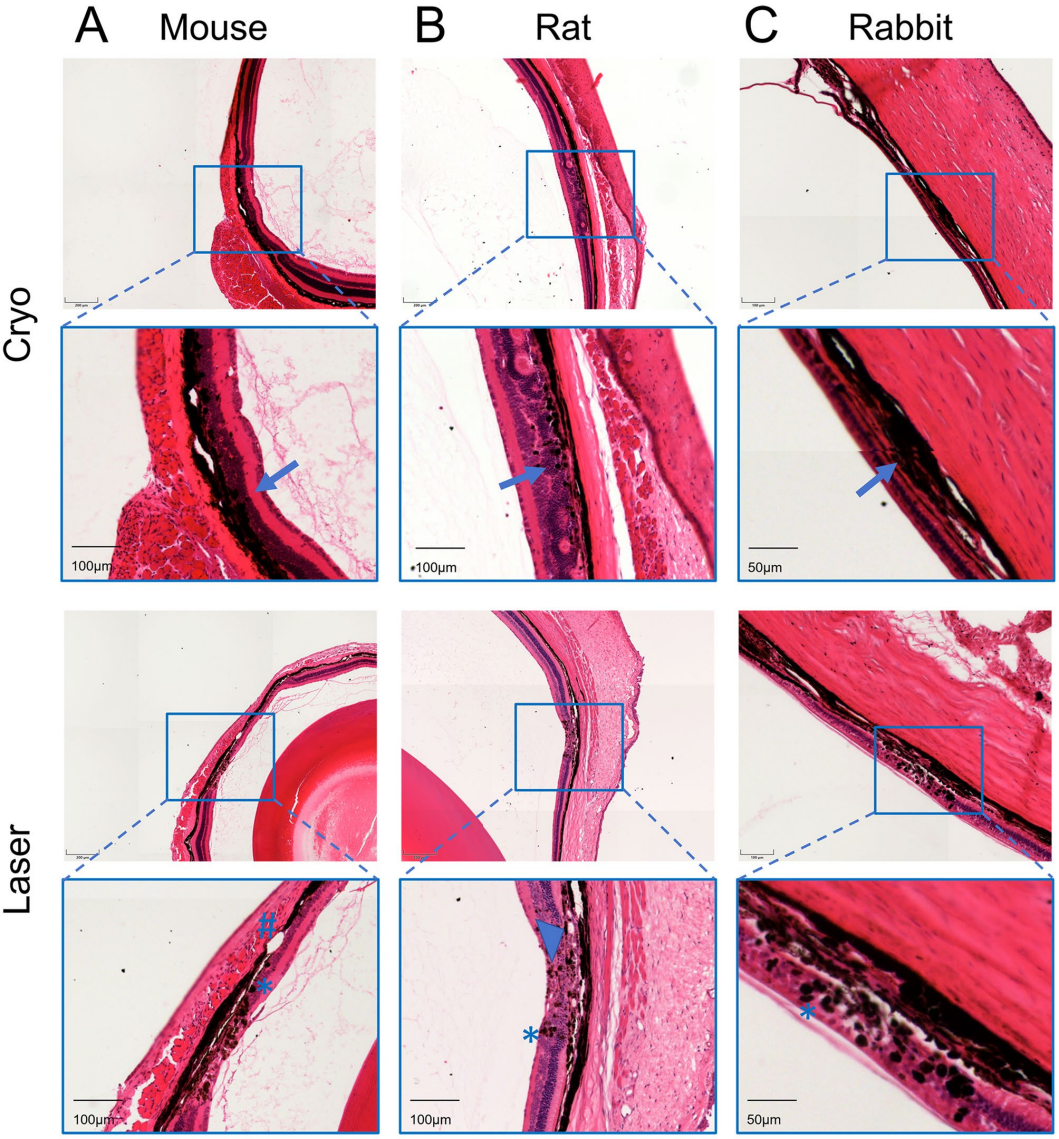
Histological effects of cryo and laser coagulation in small animal eyes. Representative histological sections of cryo (upper rows) and laser effect (lower rows) in eyes of **A** C57BL/6J mice, **B** Brown Norway rats and **C** Chinchilla Bastard rabbits using HE staining. Arrow: Folding patterns in eyes represent typical cryo coagulation lesions. Star: Examples of extensive necrosis of the nuclear elements within the lesion. Hashtag: Focal disruption of photoreceptor elements. Triangle: Vacuolation

Eyes from *wt* mice, Brown Norway rats, and Chinchilla Bastard rabbits were serially sectioned after cryo and laser coagulation, respectively, and the most optimal sagittal section focusing on the coagulation lesion was selected for reconstruction and biometric measurement (Fig. 3). All structures were well preserved, although there were still cases of common artifacts such as shallow retinal detachments.

The nuclear layers of the retina after cryo coagulation exhibited a distinct folding pattern (see Fig. 3B), accompanied by significant damage to the inner retinal layers. Following laser coagulation, there was a distinct coagulative necrosis observed in all layers of the retina. The retinal pigment epithelium (RPE) maintained its overall shape and alignment, but some of the cells exhibited vacuolization, along with a slight displacement of the pigment towards its inner wall. Both laser photocoagulation and cryotherapy applied to the anterior retina promote adhesion between the RPE and the photoreceptor (PR) layer. Observations also indicated the loss of PRs, which were replaced by a glial scar.

### 3D reconstruction of original serial sections

**Fig. 4:**
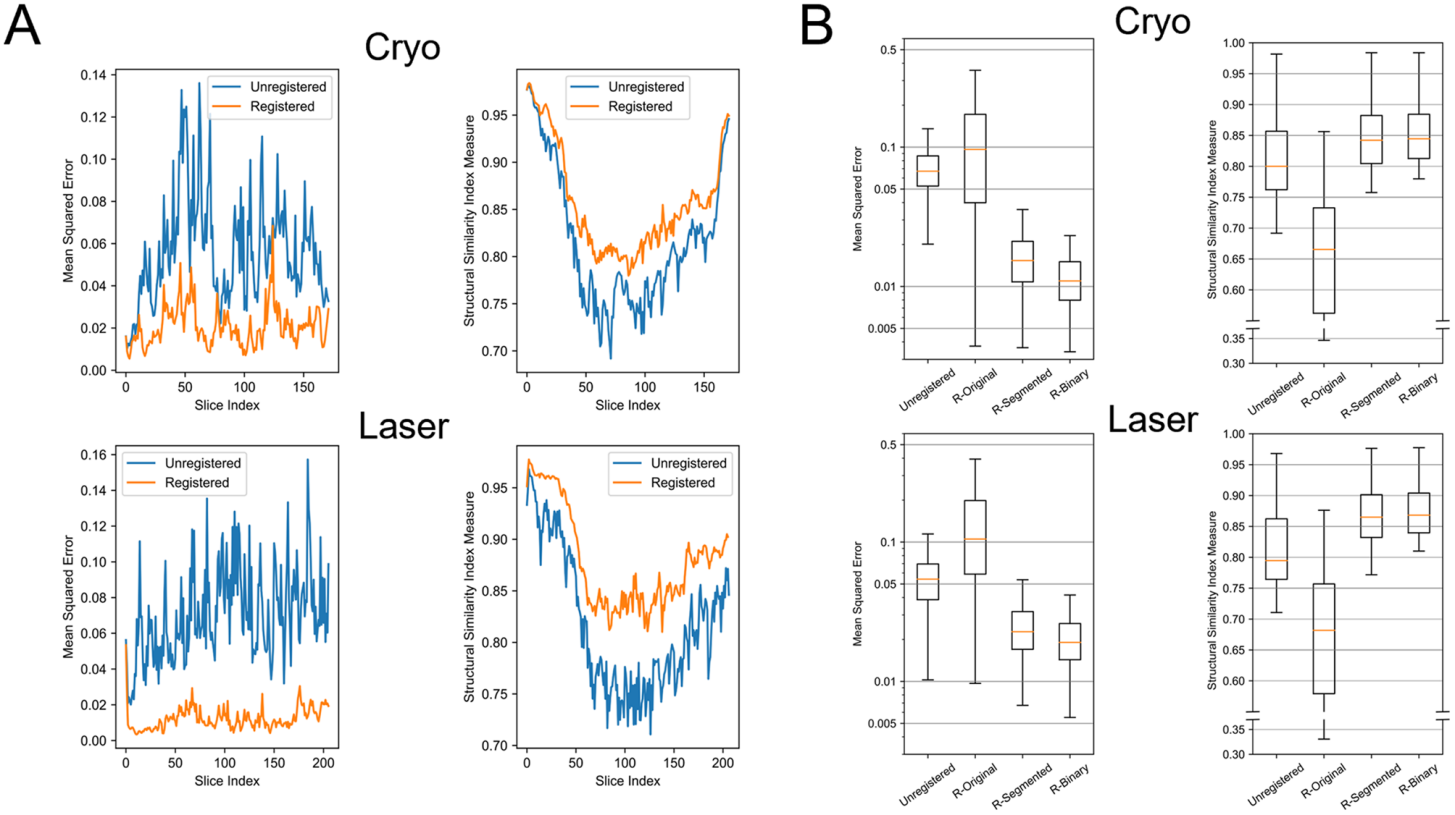
Comparison of two evaluation metrics regarding registration effects for 3D reconstruction.** A** MSE (↓*) and SSIM (↑*) in cryo and laser histological slices with or without registration (cf. R-Binary and Unregistered in Fig. 4B). The background of the slice was omitted using the SAM for the calculation of MSE and SSIM. **B** MSE (in logarithmic scale) and SSIM of histological slices in cryo and laser treated eyes with different registration approaches. Unregistered: original RGB slices were segmented without registration. R-Original: original RGB slices were first registered then segmented. R-Segmented: original RGB slices were first segmented then registered. R-Binary: original RGB slices were first segmented and binarized then registered. *An up arrow (↑) indicates that higher values of the metric are better, while a down arrow (↓) indicates that lower values are better

We created a 3D reconstruction of a mouse eye after cryo and laser coagulation as an example. Two evaluation metrics were reported, namely Mean Squared Error (MSE, the lower the better) and Structural Similarity Index Measure (SSIM, the higher the better) [10], calculated based on the moving average of four adjacent slices. We compared MSE and SSIM for registered and unregistered slices with the background omitted using the SAM (Fig. 4A) and observe improved alignment after registration (labelled R-Binary in Fig. 4B). The transformation matrix obtained from the rigid registration on the original RGB slice (R-Original), the segmented RGB slice via SAM (R-Segmented) and the binary mask of the segmentation (R-Binary) was applied to the original slice. Rigid registration without segmentation performed even worse than unregistered slices because the transformation could transcend the border due to the irregular cracks and overlaps of the slices. We found that the binary segmentation mask improved the robustness of the registration and provided the best MSE and SSIM values for both laser and cryo approaches (Fig. 4B).

**Fig. 5:**
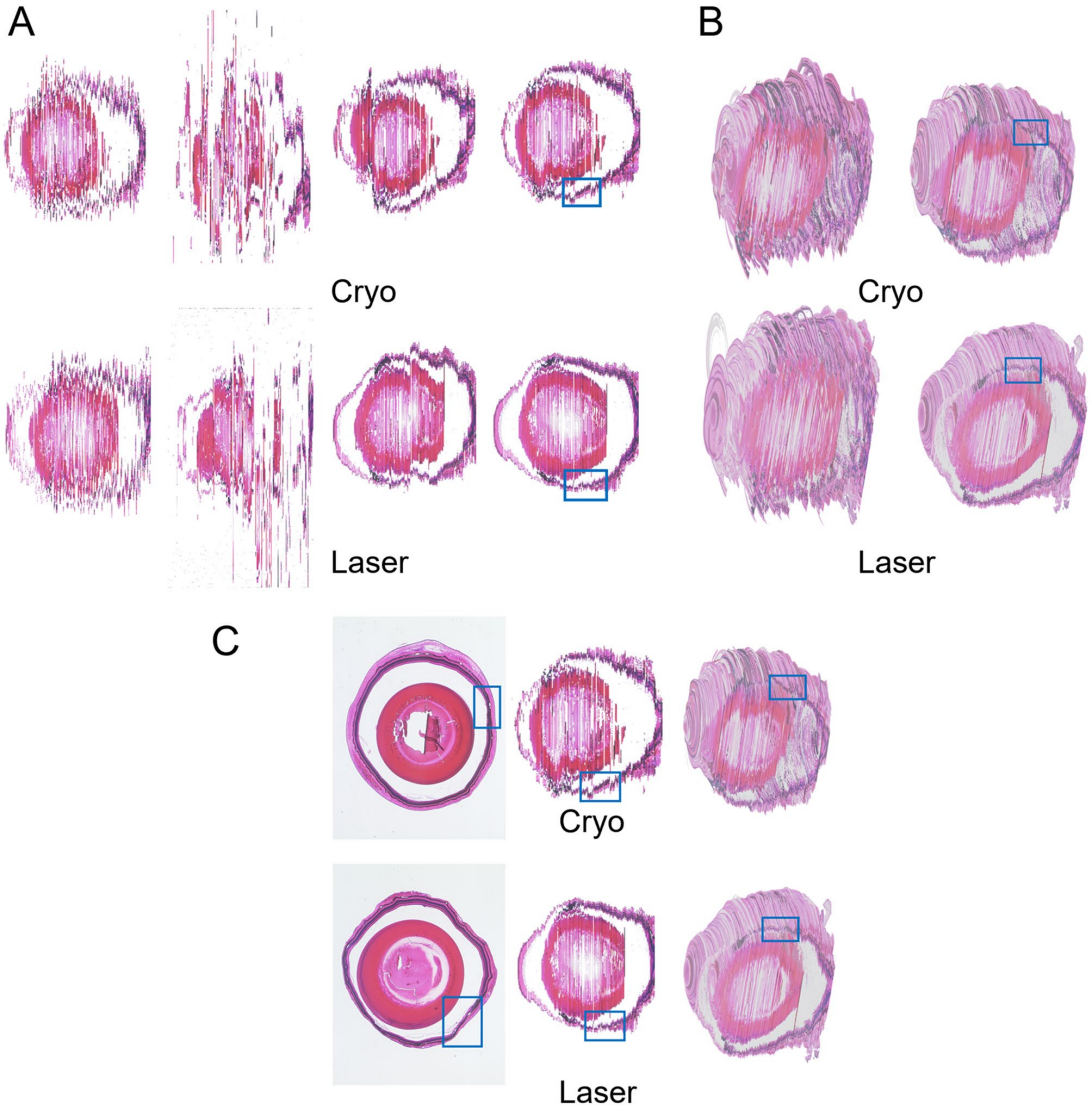
3D reconstruction of a *wt* mice eye in Blender after cryo or laser coagulation. **A** Sagittal sections for visualization of the mouse eye after cryo or laser coagulation. Cryo (from left to right): Unregistered, R-Original, R-Segmented, R-Binary. Laser (from left to right): Unregistered, R-Original, R-Segmented, R-Binary. **B** 3D reconstruction of the mouse eye after cryo or laser coagulation. Upper left: Cryo from original slices. Upper right: Cryo from registered slices. Lower left: Laser from original slices. Lower right: Laser from registered slices. **C** Post-treatment scars visualization in a mouse eye. Cryo (from left to right): original sagittal section, sagittal section of 3D reconstruction (R-Binary), 3D reconstruction. Laser (from left to right): unprocessed sagittal section of a mouse eye, sagittal section of 3D reconstruction (R-Binary), 3D reconstruction. Blue boxes indicate post-treatment scars that developed after cryo or laser procedures in the sagittal sections. Unregistered: original RGB slices were segmented without registration. R-Original: original RGB slices were first registered then segmented. R-Segmented: original RGB slices were first segmented then registered. R-Binary: original RGB slices were first segmented and binarized then registered

To this end, we stacked the aligned slices after segmentation and registration to reconstruct 3D models. Examples from the sectional view are shown in Fig. 5A. In accordance with the quantitative evaluation, the pipeline of segmentation and registration gives the best visual quality. As some manual slices were cracked or noisy, registration directly on the original images was prone to an irregular reference slice and tended to perform even worse than the original slices. Figure 5B shows the 3D reconstructed volumes with cut surfaces of the original and registered slices. To visualize the 3D modeling process and effectively demonstrate the effect of laser or cryo coagulation, Fig. 5C shows the original slices, the cross-sections of the 3D model created from these stacks and the 3D reconstruction of the model from a different angle as an example.

## Discussion

In these small series of experiments, we were able to demonstrate the ablation effects of laser and cryo treatment of the anterior retina. Because this treatment closes the preformed cleft between the RPE and the PR layer of the retina, we assume that such a pre-treatment may prevent intraoperative and insertion-related retinal detachments during experimental vitreoretinal surgery to a certain degree. The implantation of complex retinal prosthesis systems as an example may benefit from the cryo or laser induced sealing of the RPE/PR interface. However, this treatment will not prevent the risk of retinal detachments during the fixation procedure or during the manipulation of an implant inside the eye, especially in cases where implants have sharp edges as is the case in polyimide-based structures [2].

Both retinal laser therapy and cryotherapy are well-established procedures in the treatment of retinal diseases [11, 12]. Our findings on histological effects of cryo and laser coagulation in small animal eyes are consistent with those reported by Curtin et al. [13]. After coagulation, all species showed extensive necrosis of the overlying RPE and PRs, along with focal photoreceptor disruption, pigment dispersion, gliosis, and solid retinal attachment formation. We demonstrated that the pretreatment of the retina leads to the formation of scar tissue. This process may allow better access to the eye without causing retinal holes or detachments. We studied the energy dosage and placement of cryo and laser spots, outlining the core procedure and specifying key parameters. For different species, not only qualitative but also quantitative differences in effects of cryo and laser coagulation are of interest. Our data show that peripheral retinal ablation using cryo coagulation resulted in a larger lesion area and a greater decrease in retinal thickness in the eyes of mice, rats and rabbits compared to laser coagulation as expected. There was an inverse relationship between the size of the eyes and the diameter of the lesions, as well as the ratio of retinal thickness before and after coagulation. The effect of cryo coagulation in the retina was significantly more pronounced than that of laser treatment, regardless of the species. This was consistent with the observation that the diameter of the cryo probe was larger than that of the laser probe. Regarding the impact of both cryo and laser coagulation, our data indicate that increased eye size correlates with reduced lesion scale and less retinal thinning and atrophy. It is plausible to infer that larger ocular dimensions and thicker sclera might require more energy from the probe or prolong the coagulation process to achieve equivalent effects. The findings derived from animal experiments could not be directly applied to human patients with high myopia characterized by a thinner sclera and an axial elongation. Although no direct comparative studies with matched treatment parameters have yet addressed the issue, existing evidence suggests that, in high myopia, retinal scarring produced by laser coagulation tends to expand rapidly compared to emmetropic controls, hinting at a potential parallel [14]. Future studies could refine energy titration protocols by correlating in vivo scleral thickness measurements (e.g., via SS-OCT) with therapeutic outcomes in myopic patients, minimizing collateral damage while ensuring treatment efficacy. Somehow in eyes of mice and rats, the retinas after processing without cryo coagulation were slightly thicker than those without laser, despite the same histological procedure and scanning technique.

The experimental complexity differs across species. In rabbits, it was challenging to get undamaged, intact sections for histological paraffin staining and to preserve the integrity of the sections throughout the staining procedure. While evaluating experimental outcomes, not only the final data but also the practicality and complexity of the experiments should be taken into consideration to enhance our comprehension of the cross-species performance.

We created 3D models of the eyes after coagulation for mice primarily using histological images, image processing, and registration methods. These models offered a wealth of information about the eyeball, including histological tomography and tissue differentiation. By using 3D models, we can examine the cryo- and laser-induced lesions in the retina from a different and alternative perspective, helping us to improve our fundamental understanding of the effects of coagulation. The image alignment produced an original model of the true anatomical situation of the eye, in contrast to the pseudorealistic 3D model of the eyes in our previous study [9], which was generated by a full rotation of one sagittal section of the eye. We also see the possibility of transforming the 3D models into virtual reality. They offer the advantages of simulating the surgical environment in the design and methodology of animal experiments, allowing precise planning for the surgical procedure of innovative implants, as well as prior preparation for interventions.

Animal models of laser-induced retinal changes already exist, including for mice [15], rats [16], and rabbits [17], which inspired us to create a man-made scar on the anterior retina as a potentially safe insertion site for the surgical procedure. Our experimental project emphasized effects in the retinas of mice, rats, and rabbits. The rabbit eye shares many anatomical similarities with the human eye. Implantation of retinal prostheses will be carried out in a future series of experiments following this project. The decision to use mouse and rat eyes was based on the extensive development and testing of electrical stimulation and recording techniques in the retinas of these species. Furthermore, established models of genetic degeneration are currently more readily available in mice and rats. All three animal species represent an important approach to data generation and surgical planning and are therefore essential to helping human patients in the future.

The findings of this study should be interpreted with caution due to the relatively small sample size, especially for rabbits. Further studies should validate these results with larger cohorts. Lesion size was measured on a single sagittal section, which may not fully capture three-dimensional lesion morphology; nevertheless, 3D reconstructions provide qualitative volumetric context, and future studies should incorporate volumetric analyses for more accurate lesion assessment. Additionally, the results of this study are limited to observations on relatively chronic (3 weeks post-treatment) lesions. All animals may be kept for longer time frames; nonetheless, it was chosen because stabilization of therapeutic scar tissue formed by coagulation treatment in the retina is typically observed within 3 weeks. Major changes to this effect are unlikely after this period, consistent with human clinical observations [18–20]. Earlier or later changes were not captured. In addition, scleral thickness was assessed only on representative histological sections at the final endpoint, and subtle transient changes during earlier healing stages could not be excluded. Nevertheless, the shrinkage-corrected scleral thickness values observed in the present study are consistent with previously reported anatomical measurements in small animal eyes [21–24]. Future studies including multiple time points would help characterize the full temporal progression of lesions. Furthermore, the results may not have direct implications for the implantation of retinal prostheses in humans. The cryo and laser settings will show modest variations between different animal species and in comparison to the human eye. The subsequent phase of this research involves not direct clinical trials but rather testing of long-term biocompatibility and experimental implantation of complex electronic microsystems in rabbit or porcine eyes. Our findings provide foundational data on how to provide a safe implantation path for retinal stimulators in small animal eyes, representing an essential translational step for optimizing surgical procedures.

## Conclusions

Pretreatment of the anterior retina with cryo and laser coagulation may provide larger safe insertion areas in these small animal eyes, which do not have a real human-like pars plana and the latter one with a broader-based scarring formation is the preferred approach. 3D models were reconstructed from image stacks of serial sagittal eye sections, more closely resembling in-vivo anatomy, targeted planning of surgical interventions to minimize surgical complications at an early stage of an experiment.

## Data Availability

All relevant data are within the manuscript.

## **Abbreviations**

3D: Three dimensional
ONL: Outer nuclear layer
PR: Photoreceptor
RPE: Retinal pigment epithelium
SD: Standard deviation
*wt*: Wild-type
